# The Use of Anti-VDAC2 Antibody for the Combined Assessment of Human Sperm Acrosome Integrity and Ionophore A23187-Induced Acrosome Reaction

**DOI:** 10.1371/journal.pone.0016985

**Published:** 2011-02-09

**Authors:** Bianjiang Liu, Peng Wang, Zengjun Wang, Wei Zhang

**Affiliations:** Laboratory of Reproductive Medicine, Department of Urology, The First Affiliated Hospital of Nanjing Medical University, Nanjing, China; Auburn University, United States of America

## Abstract

Voltage-dependent anion channel (VDAC) is mainly located in the mitochondrial outer membrane and participates in many biological processes. In mammals, three VDAC subtypes (VDAC1, 2 and 3) have been identified. Although VDAC has been extensively studied in various tissues and cells, there is little knowledge about the distribution and function of VDAC in male mammalian reproductive system. Several studies have demonstrated that VDAC exists in mammalian spermatozoa and is implicated in spermatogenesis, sperm maturation, motility and fertilization. However, there is no knowledge about the respective localization and function of three VDAC subtypes in human spermatozoa. In this study, we focused on the presence of VDAC2 in human spermatozoa and its possible role in the acrosomal integrity and acrosome reaction using specific anti-VDAC2 monoclonal antibody for the first time. The results exhibited that native VDAC2 existed in the membrane components of human spermatozoa. The co-incubation of spermatozoa with anti-VDAC2 antibody did not affect the acrosomal integrity and acrosome reaction, but inhibited ionophore A23187-induced intracellular Ca^2+^ increase. Our study suggested that VDAC2 was located in the acrosomal membrane or plasma membrane of human spermatozoa, and played putative roles in sperm functions through mediating Ca^2+^ transmembrane transport.

## Introduction

Voltage-dependent anion channel (VDAC), as a membrane channel protein, is firstly identified in the mitochondrial outer membrane of *Paramecium Aurelia* [1], [2]. It has now been discovered in the mitochondrial outer membrane of most eukaryotes [3]. VDAC is highly conserved in molecular structure and function during evolution [4], [5]. In mammals, three homologous genes encode and express three corresponding protein subtypes with similar molecular weight (30–35 kDa), each of them shares approximately 70% identity to the others [4]–[6]. Current studies show that the most abundant subtype is VDAC1 and that the least common form is VDAC3 [7], [8]. VDAC1 and VDAC2 can form the channel structure across the artificial lipid bilayer in vitro, but VDAC3 does not easily incorporate in the reconstituted membrane [9]. VDAC in the mitochondrial outer membrane can regulate membrane permeability to small ions and molecules (e.g. Na^+^, Ca^2+^, Cl^−^, ATP, glutamate) according to membrane potential changes [10]–[13]. Therefore, VDAC is reportedly involved in many mitochondria-related biological processes, such as energy metabolism and cell apoptosis [14]–[17]. VDAC is once thought to be only localized in the mitochondrial outer membrane [18], [19]. However this protein is recently found in the plasma membrane or other non-mitochondrial cellular components, which implies that VDAC has more novel functions [20]–[22].

Although VDAC has been extensively studied in various tissues and cells, there is little knowledge about the distribution and function of VDAC in male mammalian reproductive system. According to current animal studies, VDAC1 is exclusively localized in the Sertoli cells, and VDAC2 and VDAC3 are present in the germ cells [23]–[25]. In mature spermatozoa, VDAC2 and VDAC3 are abundant in the outer dense fibers of flagellum, a non-membranous structure [26]. VDAC2 is also found in the acrosomal membrane or plasma membrane of sperm head [27]. Functionally, VDAC is implicated in spermatogenesis, sperm maturation, motility and fertilization [28]. However, the exact localization and function of three VDAC subtypes in mammalian spermatozoa have not yet been established.

Mammalian spermatozoa are a kind of highly compartmentalized cells. Proteins involved in the acrosomal status and acrosome reaction are usually located in the head or acrosomal region. The intact acrosome is a prerequisite for normal acrosome reaction and sperm-egg fusion [29]. It is now generally agreed that acrosome reaction is a Ca^2+^-dependent event [30]. The occurrence of acrosome reaction has a positive correlation with intracellular Ca^2+^ concentration. Acrosome reaction can therefore be induced through co-incubation of spermatozoa with calcium ionophore A23187 in vitro [31], [32]. VDAC2 has been discovered in the acrosomal membrane or plasma membrane of bovine sperm head [27]. The co-incubation of bovine spermatozoa with anti-VDAC2 antibody can cause an increased loss of acrosomal integrity and noticeable changes in the morphology of sperm head, which are presumably due to the alteration of the intracellular ion concentration [27]. VDAC in somatic cells contains Ca^2+^ binding site and regulates Ca^2+^ transmembrane transport [33], [34]. These data prompt us to hypothesize that VDAC2 incorporates in the sperm membrane and regulates the acrosomal integrity and acrosome reaction through mediating Ca^2+^ transmembrane flux, a typical feature of VDAC as a membrane channel protein. In a previous study, we have confirmed the presence of VDAC in human spermatozoa [35]. Up to now, there is no knowledge about the respective distribution and function of three VDAC subtypes in human spermatozoa. The purpose of this study is to study the presence of VDAC2 in human spermatozoa for the first time, and to investigate its functional role in the acrosomal integrity and acrosome reaction using anti-VDAC2 monoclonal antibody.

## Methods

Approval for this study was granted by the ethics committee of Nanjing Medical University (China) prior to sample collection and informed written consent was received from all participants of this study. All chemicals and reagents used in this study were molecular biology grade purchased from Sigma-Aldrich (St. Louis, MO, USA) unless otherwise stated.

### 2.1. Generation of anti-VDAC2 monoclonal antibody

The recombinant full length human VDAC2 protein used as the antigen was expressed and purified according to our previous protocol [35]. The mouse anti-human VDAC2 monoclonal antibody was produced by Genscript (Piscataway, NJ, USA). Antibody binding activity and specificity were evaluated by ELISA. Affinity purification of antibody was performed using recombinant VDAC2 protein and Protein A/G.

### 2.2. Semen preparation

Freshly ejaculated human semen samples were obtained from donors by masturbation after 3–7 days of sexual abstinence. All samples had normal semen parameters according to World Health Organization guidelines [36]. The liquefied ejaculate was washed on a two-layer (90% and 45%) discontinuous Percoll (GE Healthcare, Piscataway, NJ, USA) gradient. After centrifugation at 400 g for 18 min, the cell pellet from the 90% layer was washed twice in Biggers-Whitten-Whittingham medium (BWW; 95 mM NaCl/44 µM sodium lactate/25 mM NaHCO_3_/20 mM Hepes/5.6 mM D-glucose/4.6 mM KCl/1.7 mM CaCl_2_/1.2 mM KH_2_PO4/1.2 mM MgSO_4_/0.27 mM sodium pyruvate/0.3% wt/vol BSA, 5 units per ml penicillin/5 µg/ml streptomycin, pH 7.4). The 2 ml BWW medium was layered over the pellet, and spermatozoa were allowed to “swim up” into the medium in a 5% CO_2_ incubator. After 1 h, the BWW medium supernatant layer was removed and centrifuged at 400 g for 5 min to collect the spermatozoa.

### 2.3. Western blot analysis

Sperm pellets prepared as above were resuspended in lysis buffer (50 mM Tris, 150 mM NaCl, 1% Triton X-100, 1% sodium deoxycholate, 0.1% SDS, 2 mM Na_3_VO_4_, 50 mM NaF, 10 µg/ml leupeptin) containing a Halt Protease Inhibitor Cocktail (Thermo Scientific, Rockford, IL, USA) for 2 h at 4°C, sonicated for 1 min on ice, and centrifugated at 12000 g for 15 min at 4°C. The extraction of hydrophobic membrane protein was performed as described previously [37]. Briefly, the supernatant was extracted with chloroform and methanol (v/v = 3∶1) and centrifugated at 12000 g for 10 min at 4°C. The organic-aqueous interface was dissolved in 4 M urea and stored at −20°C until use. Three recombinant human VDAC proteins were acquired according to our previous report [35]. The protein concentration was determined by Bicinchoninic Acid assay. Aliquots of protein samples were boiled in sodium dodecyl sulphate (SDS) sample buffer and resolved by SDS-PAGE followed by transfer onto a Polyvinylidene Fluoride (PVDF) membrane (Bio-Rad, Hercules, CA, USA). The membrane was blocked with 5% non-fat milk in Tris-buffered saline (TBS; pH 7.4) for 2 h before being incubated with anti-VDAC2 antibody diluted in blocking solution at 4°C overnight. The membrane was then washed three times in TBS and probed with horseradish peroxidase (HRP) conjugated goat anti-mouse IgG (1∶5000; Zhongshan Goldenbridge Biotechnology, Beijing, China) at 37°C for 1 h. After washing, an ECL reaction kit (Cell Signaling Technology, Danvers, MA, USA) was employed to detect the peroxidase activity and images were captured by FluorChem 5500 (Cell Biosciences, Santa Clara, CA, USA).

### 2.4. Assessment of acrosomal status

Spermatozoa collected as described above were adjusted to 2×10^6^ cells/ml in BWW and incubated with different dilutions of anti-VDAC2 antibody in a 5% CO_2_ incubator. Control experiments were included: anti-VDAC2 antibody was omitted or replaced by normal mouse IgG. After incubation, sperm suspensions were air-dried onto poly-lysine-coated slides and fixed with 4% paraformaldehyde in Phosphate-buffered saline (PBS; pH 7.6) for 1 h at 4°C. After washing, slides were incubated with 25 µg/ml fluorescein isothiocyanate FITC-labeled pisum sativum agglutinin (FITC-PSA; Sigma-Aldrich, St. Louis, MO, USA) in dark room for 1 h at room temperature. Then slides were washed and mounted with VECTASHIELD® Mounting Medium (Vector Laboratories, Burlingame, CA, USA) to avoid fluorescence fading. The acrosomal status was evaluated under an Axioskop 2 plus fluorescent microscope (Carl Zeiss, Thornwood, NY, USA) with ×1000 magnification. At least 200 spermatozoa were counted in each smear. The counts were scored blindly and counted in duplicate slides.

### 2.5. Assessment of acrosome reaction

Spermatozoa collected as described above were adjusted to 2×10^6^ cells/ml in BWW and incubated for 2 and 4 h. Sperm suspensions were loaded with different dilutions of anti-VDAC2 antibodies, normal mouse IgG, or without any antibody, and then were divided into two aliquots. One aliquot was exposed to calcium ionophore A23187 (10 µm final concentration; Sigma-Aldrich, St. Louis, MO, USA) to induce acrosome reaction and the other was given no treatment as the control for spontaneous acrosome reaction. Both were incubated at 37°C, 5% CO_2_ and 95% air for 30 min. After incubation, acrosome reaction was evaluated and counted using the FITC-PSA staining as described above.

### 2.6. Measurement of intracellular Ca^2+^ during acrosome reaction

A kinetic intracellular Ca^2+^ concentration during ionophore A23187-induced acrosome reaction was measured by an Infinite M200 Microplate Reader (Tecan Group, Männedorf, Switzerland). Briefly, after incubation as described above, sperm suspensions were loaded with a fluorescent calcium probe Fluo-3 AM (5 µM final concentration; Invitrogen, Carlsbad, CA, USA) at 37°C for 30 min and then washed twice at 400 g for 5 min to remove free fluo-3 AM. Fluo-3 AM-loaded spermatozoa were resuspened in BWW and exposed to A23187 to induce acrosome reaction. The time course evaluation of the intracellular Ca^2+^ fluorescence signal was recorded by the Microplate Reader. The instrument was preheated to 37°C and fluorescence intensity was read at intervals of 15 s. The fluorescence of Fluo-3 was excited at 488 nm and measured with a 530 nm filter. The measurement lasted for 22 min, containing an initial 2 min of control period before A23187 were added. The changes of fluorescence intensity were analyzed as previously reported [38].

### 2.7. Statistical analysis

Data were expressed as means ± standard error of the mean (SEM). An arcsine square-root transformation was performed on percentage data. Differences between experimental groups were assessed by one-way or two way analysis of variance. Statistically significant differences were determined at P<0.05.

## Results

### 3.1. Confirmation of antibody specificity and native VDAC2 in human spermatozoa

**Figure 1:**
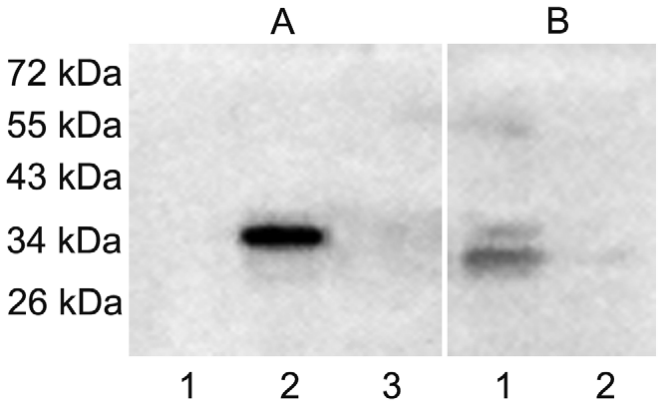
Antibody specificity and native VDAC2 in human spermatozoa analyzed by western blot. A: recombinant VDAC1 (lane 1), VDAC2 (lane 2) and VDAC3 (lane 3) proteins were probed with anti-VDAC2 antibody. B: the hydrophobic membrane protein extracts from human spermatozoa were separated by SDS-PAGE and probed with anti-VDAC2 antibody (lane 1) or the same antibody pre-absorbed with an excess of recombinant VDAC2 protein. The position of molecular weight standards (kDa) is indicated on the left.

To firstly assess the specificity of anti-VDAC2 monoclonal antibody used in our study, we observed the antibody reaction to three recombinant human VDAC proteins derived from the testis. As shown in Fig. 1A, the antibody only recognized VDAC2. Then the hydrophobic membrane protein extracts from purified human spermatozoa were separated by SDS-PAGE and probed with specific anti-VDAC2 antibody. As shown in Fig. 1B, lane 1, the approximately 30 kDa native VDAC2 was discovered. As the control, no protein bands were observed using the same antibody pre-absorbed with an excess of recombinant VDAC2 protein (Fig. 1B, lane 2).

### 3.2. Effect of anti-VDAC2 antibody on acrosomal integrity

**Figure 2:**
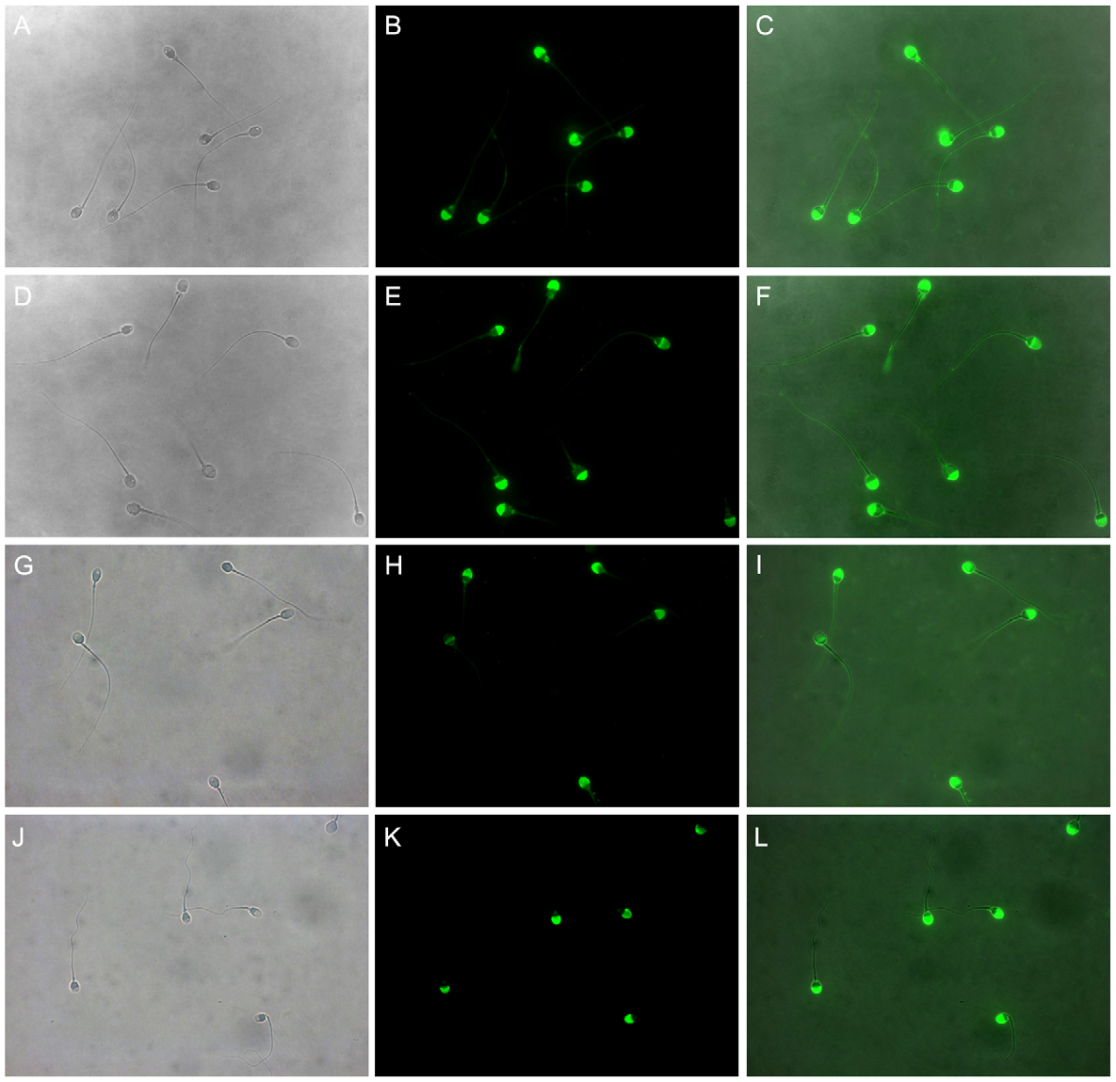
FITC-PSA staining of human spermatozoa in different treatment groups. A–C: spermatozoa were separated and stained immediately. D–F: spermatozoa were incubated with anti-VDAC2 antibody. G–I: spermatozoa were incubated with normal mouse IgG. J–L: spermatozoa were incubated without any antibody. A, D, G and J: phase-contrast images; B, E, H and K: immunofluorescent images; C, F, I and L: merged images. Magnification was ×1000.

**Figure 3:**
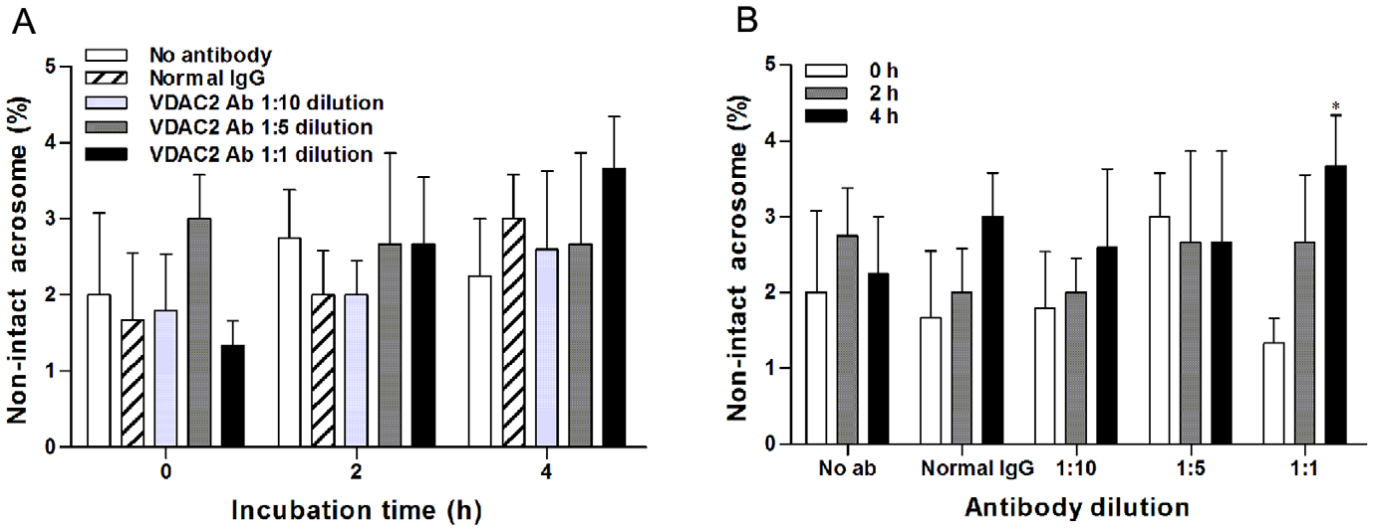
Effect of anti-VDAC2 antibody on acrosomal integrity. A: spermatozoa were incubated with normal mouse IgG or anti-VDAC2antibody at different dilutions and counted according to the different incubation time. B: spermatozoa were incubated for the different time and counted according to different treatments. At least 200 spermatozoa were counted in each sample. Data were shown as mean ± SEM of six experiments; *, P < 0.05 versus control.

The acrosomal status of human spermatozoa after incubation with anti-VDAC2 antibody was checked by FITC-PSA staining. As shown in Fig. 2, A–C, almost all the separated spermatozoa remain intact acrosomes when they were stained immediately. After 2 or 4 h of incubation with anti-VDAC2 antibody, only few spermatozoa showed non-intact acrosomes (Fig. 2, D–F). Similar images appeared when spermatozoa were incubated with normal mouse IgG (Fig. 2, G–I) or without any antibody (Fig. 2, J–L). Except for 0 h versus 4 h at 1∶1 dilution (P = 0.045), there were no significant differences in the percentage of non-intact acrosomes between different treatment groups (Fig. 3).

### 3.3. Effect of anti-VDAC2 antibody on acrosome reaction

**Figure 4:**
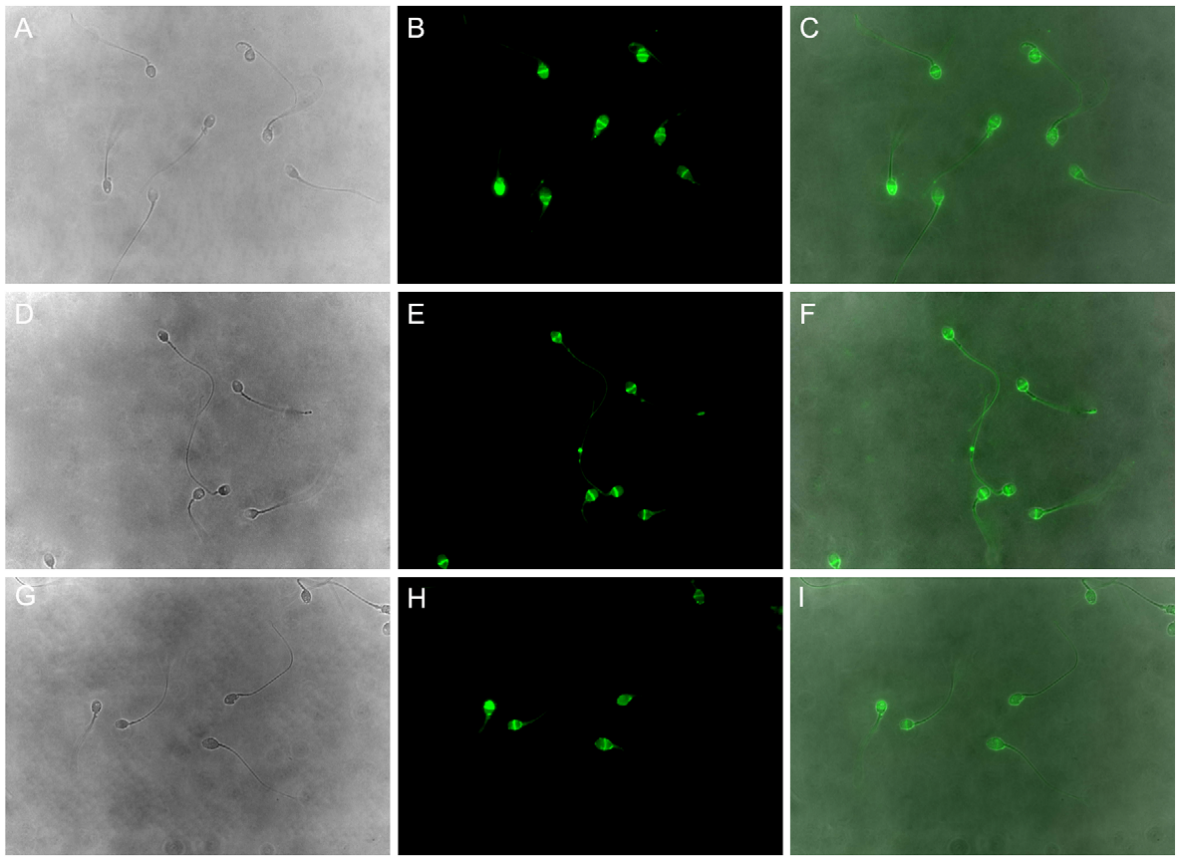
FITC-PSA staining of human spermatozoa in different treatment groups. A–C: spermatozoa were loaded with anti-VDAC2 antibody and induced by A23187. D–F: spermatozoa were loaded with normal mouse IgG and induced by A23187. G–I: spermatozoa were not loaded with any antibody and induced by A23187. A, D and G: phase-contrast images; B, E and H: immunofluorescent images; C, F and I: merged images. Magnification was ×1000.

**Figure 5:**
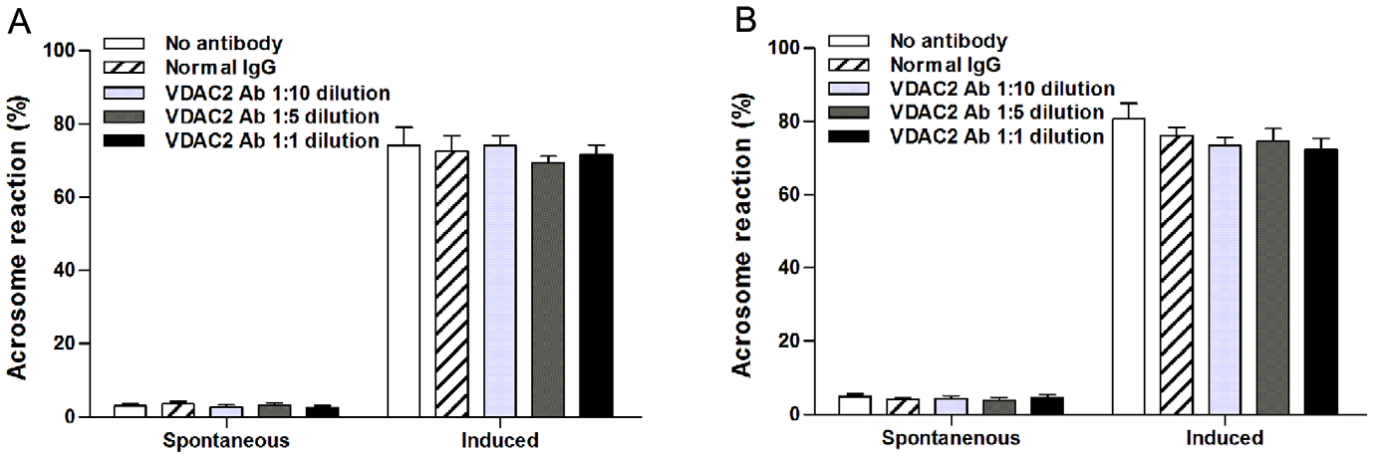
Effect of anti-VDAC2 antibody on acrosome reaction. A: spermatozoa were incubated for 2 h, loaded with normal mouse IgG or anti-VDAC2antibody at different dilutions, and counted with or without A23187. B: spermatozoa were incubated for 4 h, loaded with normal mouse IgG or anti-VDAC2antibody at different dilutions, and counted with or without A23187. At least 200 spermatozoa were counted in each sample. Data were shown as mean ± SEM of six experiments.

To investigate the effect of anti-VDAC2 antibody on acrosome reaction, ionophore A23187 was loaded to induce acrosome reaction and the acrosome reaction rate was calculated using FITC-PSA staining. As shown in Fig. 4, acrosome reaction occurred in most spermatozoa whether or not they were incubated with anti-VDAC2 antibody. Although the percentage of acrosome reaction induced by A23187 increased obviously than that of spontaneous acrosome reaction, there were no statistically significant differences between different treatment groups (Fig. 5).

### 3.4. Effect of anti-VDAC2 antibody on A23187-induced intracellular Ca^2+^ concentration

**Figure 6:**
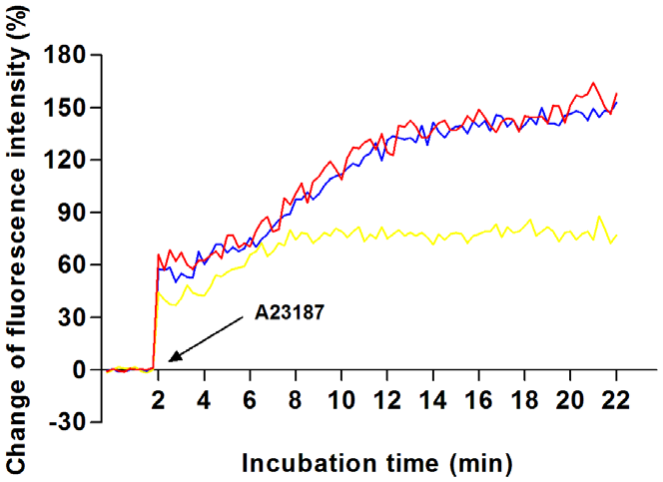
Effect of anti-VDAC2 antibody on A23187-induced Ca^2+^ influx. Spermatozoa were incubated for 2 h, then loaded with anti-VDAC2 antibody (yellow), normal mouse IgG (blue), or without any antibody (red), and finally stimulated by A23187. The changes of fluorescence intensity reflecting intracellular Ca^2+^ concentration were recorded by the Microplate Reader.

In order to further assess the effect of anti-VDAC2 antibody on Ca^2+^ transmembrane transport, intracellular Ca^2+^ concentration was recorded during artificial A23187-induced acrosome reaction. Fluo-3 AM was loaded in sperm suspensions and the fluorescence intensity reflecting intracellular Ca^2+^ concentration was measured by the Microplate Reader. Fig. 6 showed the changes of intracellular Ca^2+^ fluorescence signal. After spermatozoa were incubated for 2 h and loaded with normal mouse IgG or without any antibody, the fluorescence intensity elevated sharply once A23187 was added, then showed a sustained and sluggish elevation, and finally kept stable. When anti-VDAC2 antibody (1∶1 dilution) was added, the elevation was inhibited. Similar image appeared after spermatozoa were incubated for 4 h and measured (not shown).

## Discussion

Although there are several researches about VDAC in male mammalian testis and spermatozoa, the respective presence and function of three VDAC subtypes have not yet been established. In the present study, we focused on the presence and functional role of VDAC2 in human spermatozoa. The binding specificity of anti-VDAC2 antibody used in our study was firstly confirmed. Native VDAC2 protein was identified in the hydrophobic membrane protein extracts from human spermatozoa. Our results suggested that VDAC2 was located in membrane components. The anti-VDAC2 antibody will contribute to our further study about the new characteristics and functions of VDAC2 in human spermatozoa.

It has been demonstrated that VDAC2 can form the channel structure in the lipid bilayer and play important roles in cellular functions through mediating the transmembrane ion transport. A recent study reported that the co-incubation of bovine spermatozoa with anti-VDAC2 antibody led to the time- and dose-dependent destruction of acrosomal integrity [27]. Presumably, this was because the binding and interaction of antibody with VDAC2 interfered with VDAC-mediated ion transport. However, similar results did not appear in our study despite using a similar method. The acrosomal integrity did not show significant changes between different treatment groups. Different antibodies and species might lead to these contrasts. Another possibility is that VDAC2 exists only in the acrosomal membrane of human spermatozoa and fails to be recognized effectively by the antibody. In our further research, we will study the more accurate localization of VDAC2 at the ultrastructural level.

The acrosome is a cap-like vesicle under the plasma membrane of sperm head. During acrosome reaction, acrosomal outer membrane and proximate plasma membrane fuse together. Subsequently, the membrane permeability is increased, followed by destructed acrosomal integrity and acrosomal exocytosis. Acrosome reaction is a complex and irreversible process, with many ions, molecules and signaling pathways involved in [39], [40]. Although the regulatory mechanism remains unclear, acrosome reaction is generally considered to be Ca^2+^-dependent [30]. Since VDAC in somatic cells can mediate Ca^2+^ transport, we further investigate the possible role of VDAC2 in A23187-induced Ca^2+^ flux and acrosome reaction. Our data revealed that A23187 evidently promoted the concentration of intracellular Ca^2+^ and the incidence of acrosome reaction. When specific anti-VDAC2 antibody was added, intracellular Ca^2+^ increase induced by A23187 was inhibited. Our present study demonstrated that VDAC2 participated in the mediation of Ca^2+^ transmembrane transport, and the co-incubation of human spermatozoa with anti-VDAC2 antibody inhibited A23187-induced Ca^2+^ flux. During acrosome reaction, the fusion and destruction of acrosomal membrane and plasma membrane might promote the binding and interaction of antibody with VDAC2. It then interfered with the permeability of VDAC channel to Ca^2+^ during the fusion of acrosomal membrane and plasma membrane. It is worth noting that the relationship between VDAC channel states and its permeability to Ca^2+^ remains controversial. A high quality research has reported that VDAC closure increases Ca^2+^ flux [41]. However this is a direct contrast to others who observe that VDAC opening promotes Ca^2+^ flux into the mitochondrial membrane [33]. Therefore the exact mechanism of anti-VDAC2 antibody affecting VDAC-mediated Ca^2+^ transmembrane transport in human spermatozoa needs further study. We also note that anti-VDAC2 antibody didn't affect acrosome reaction rate evidently, although the co-incubation inhibited the intracellular Ca^2+^ increase induced by A23187. It is generally believed that human spermatozoa possess different types of Ca^2+^ permeable channels with different functional properties [42]–[45]. The Ca^2+^ transport mediated by other Ca^2+^ permeable channels might compensate for the inhibition of anti-VDAC2 antibody. Thus there is no significant change in the eventual acrosome reaction rate. Additionally, intracellular Ca^2+^ increase is mainly derived from two sources during acrosome reaction: influx of extracellular Ca^2+^ and release of intracellular calcium stores [46]. Which regulatory mechanism was dominant in A23187-induced acrosome reaction, and which regulatory pathway anti-VDAC2 antibody was involved in, all needed further study.

In summary, this study demonstrates the presence of VDAC2 in the membrane components of human spermatozoa and exhibits the functional role of VDAC2 on A23187-induced intracellular Ca^2+^ increase during acrosome reaction. Our study yields the first clue for the participation of VDAC2 in Ca^2+^ transmembrane transport in human spermatozoa. Ca^2+^ and other ions (such as Na^+^, Cl^−^ and HCO_3_^−^) have been validated to play important roles in sperm motility, capacitation and sperm-egg fusion. It is interesting to research whether VDAC2 is implicated in these sperm functions through mediating corresponding ions. Accordingly, more in-depth studies are required to investigate the ultrastructural localization, the new functional roles, and the accurate molecular mechanism of VDAC2 in human spermatozoa.
